# Exploring the Impact of Toxic Attitudes and a Toxic Environment on the Veterinary Healthcare Team

**DOI:** 10.3389/fvets.2015.00078

**Published:** 2015-12-23

**Authors:** Irene C. Moore, Jason B. Coe, Cindy L. Adams, Peter D. Conlon, Jan M. Sargeant

**Affiliations:** ^1^University of Guelph, Ridgetown, ON, Canada; ^2^Department of Population Medicine, Ontario Veterinary College, University of Guelph, Guelph, ON, Canada; ^3^Faculty of Veterinary Medicine, University of Calgary, Calgary, AB, Canada; ^4^Hill’s Pet Nutrition Primary Healthcare Centre, Ontario Veterinary College, University of Guelph, Guelph, ON, Canada

**Keywords:** veterinary, workplace behavior, organizational behavior, communication, leadership effectiveness

## Abstract

The objective of this qualitative study was to compare veterinarians’ and Registered Veterinary Technicians’ (RVT’s) perceptions of the veterinary healthcare team with respect to the impact of toxic attitudes and a toxic environment. Focus group interviews using a semi-structured interview guide and follow up probes were held with four veterinarian groups (23 companion animal veterinarians) and four Registered Veterinary Technician groups (26 RVTs). Thematic analysis of the discussions indicated both veterinarian and RVT participants felt team members with manifestations of toxic attitudes negatively impacted veterinary team function. These manifestations included people being disrespectful, being resistant to change, always wanting to be the “go to person,” avoiding conflict, and lacking motivation. When conflict was ignored, or when people with toxic attitudes were not addressed, a toxic environment often resulted. A toxic environment sometimes manifested when “broken communication and tension between staff members” occurred as a result of employees lacking confidence, skills, or knowledge not being managed properly. It also occurred when employees did not feel appreciated, when there was difficulty coping with turnover, and when there were conflicting demands. The presence of people manifesting a toxic attitude was a source of frustration for both veterinarian and RVT participants. Prompt and consistent attention to negative behaviors is recommended to reduce the development of a toxic environment.

## Introduction

The use of teams in human healthcare to coordinate work has been promoted since the beginning of the twentieth century. The effectiveness of this model is being increasingly researched (1, 2). Although the team approach to veterinary medicine has been advocated for decades (3–5), empirical research on the topic is sparse. Teams are generally considered across disciplines to consist of individuals with interdependent tasks, sharing responsibilities for outcomes (2, 6). Characteristics of cohesive teams include having clear, measurable goals, trained team members, division of labor, and effective communication (7). Research in the human health field has shown that successfully functioning teams with effective communication lead to better quality of care with enhanced patient outcomes (e.g., reduced post-operative pain, improved post-operative functioning, shorter hospital stay, improved patient ratings of care) (8–12) and improved job satisfaction (13). Conversely, medical errors often result from miscommunication within malfunctioning human healthcare teams (14, 15). Furthermore, in human hospitals, unfavorable nursing practice environments have been associated with job dissatisfaction, emotional exhaustion, intent to leave, and fair to poor quality patient care (16).

The impact of employees with negative behaviors and a negative work environment has been described in the veterinary press for many years, yet veterinary practices continue to be plagued with the recurring issues that cause these situations (17–19). Communication problems between veterinarians and with staff contribute to lower job satisfaction for veterinarians (20, 21) and affect job satisfaction and incidence of burnout in other members of the veterinary healthcare team (22).

The objective of this study was to compare veterinarians’ and Registered Veterinary Technicians’ (RVT’s) perceptions of the veterinary healthcare team. Part of a study on team effectiveness, this paper focuses on the impact of toxic attitudes and a toxic environment on veterinary team function. Since limited research has been conducted in this area, an inductive research approach was utilized. Through the use of focus groups, participants were provided an opportunity to share their experiences working within healthcare teams, including identifying factors which may enhance or detract from optimal team function. The use of focus groups in qualitative research provides data in the words of the participants, which is then analyzed to identify patterns and trends (23). Focus groups have been increasingly used in human and veterinary healthcare research (24–27).

By discussing ideas, experiences, and perceptions in a small group setting, this study allowed participants to reflect on their own and their coworkers roles in the veterinary team. Findings will enhance team function within private companion animal practice and assist in future development of best practice veterinary team guidelines.

## Materials and Methods

The study protocol was approved by the University of Guelph Research Ethics Board (REB#09MR008). Four independent veterinarian and four independent technician focus groups were conducted to explore perceptions of the veterinary team.

### Study Participants

Using the publicly accessible College of Veterinarians (CVO) database, the sampling frame consisted of all veterinarians designated as small animal practitioners from eight counties (Brandt, Halton, Hamilton-Wentworth, Oxford, Peel, Perth, Waterloo, and Wellington) in the Province of Ontario. This area contains approximately 13% of all veterinarians registered in the province and is within approximately 2 h of the Ontario Veterinary College. A random number generator was utilized to identify potential participants from the sampling frame. For the veterinarian focus groups, initial contact was made by a letter of introduction mailed to each randomly selected veterinarian outlining the study and offering dinner and a $40.00 honorarium for participation. One to 2 weeks later, a follow up phone call was made by the author (Irene C. Moore) to address questions and obtain initial consent.

Registered Veterinary Technicians were recruited from the Ontario Association of Veterinary Technicians (OAVT) data base, utilizing postal codes from the same geographic area as the veterinarian groups, using a random number generator. Since the RVT data base is not publicly accessible, the OAVT mailed a letter of invitation on the researchers’ behalf. The mailing included a letter of introduction outlining the study and offering dinner and a $40.00 honorarium for participation. Members interested in participating were then required to contact the author (Irene C. Moore) by phone or e-mail. A reminder letter was sent via the OAVT 2 weeks after the additional mailing. Letters were sent out to RVTs in the sampling frame until each focus group had a minimum of four participants.

Reminder emails and phone calls were made to both veterinarian and RVT participants 1–2 days prior to their scheduled focus group session. Two focus group meetings for each cohort (i.e., veterinarian and RVT) were held in a hotel conference room in Kitchener, ON, Canada, and similarly two for each group were held in Mississauga, ON, Canada. Each focus group lasted approximately 2 h. The technician focus group meetings took place in June 2009, while the veterinarian meetings took place in September 2009.

### Focus Group Interviews

After informed consent forms were signed, the focus group meetings were conducted by a professional moderator with extensive experience conducting focus groups, and a veterinary student assistant. Informed consent forms included an assurance that every effort would be taken to ensure participant confidentiality of information shared during the study. Semi-structured discussion guides developed by two of the authors (Irene C. Moore and Jason B. Coe) were used (Appendices I and II in Supplementary Material). Participants were asked primarily open-ended questions regarding their perspectives on the veterinary healthcare team, and how their role fit within the team. They were also asked about their responsibilities in that role and about their interactions with other team members. Finally, participants were asked to describe the potential benefits and challenges encountered in working in a veterinary team environment.

All sessions were digitally audio-recorded and subsequently transcribed verbatim by the moderator and confirmed for accuracy by the veterinary student assistant.

### Data Analysis

**Figure 1:**
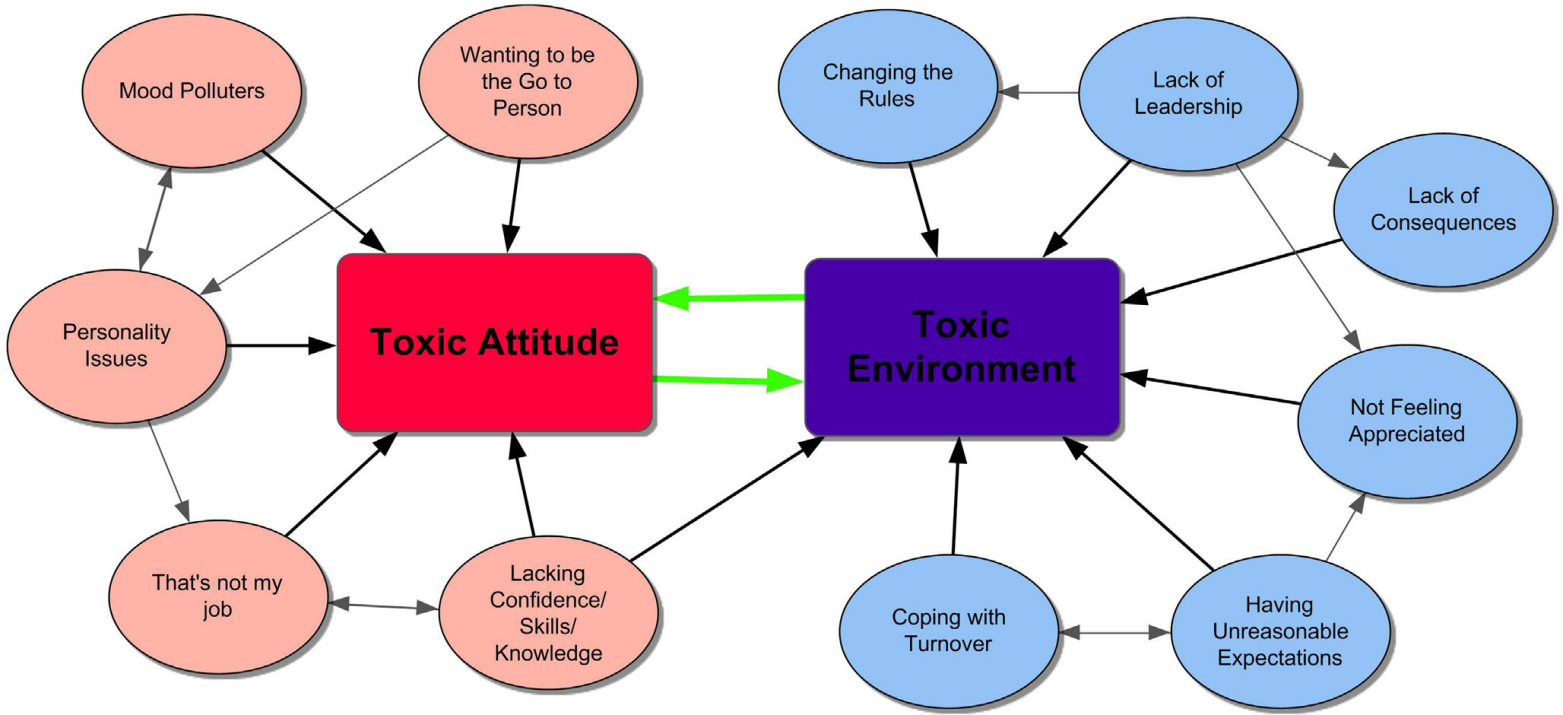
**Thematic map of the interrelationships of toxic attitude and toxic environment**.

Using the verbatim transcripts, the focus group discussions were analyzed by the first author (Irene C. Moore) using thematic analysis, a qualitative analytic method. Thematic analysis is “a method for identifying, analyzing, and reporting patterns (themes) within data” (28). The transcripts were reviewed a number of times both by listening to the recordings and reading the written transcripts. An inductive approach was then used to develop themes (29), looking for trends and patterns common to the various focus groups. A number of themes emerged, with the initial codes constantly compared with developing ones. Some themes were identified only in RVT groups and others only in veterinarian groups. Themes and subthemes were reviewed repeatedly to determine whether themes could be collapsed or refined. After construction of a thematic map (Figure 1), possible interrelationships among the themes were examined. Intercoder reliability was assessed by having an RVT and a veterinarian not involved in the focus groups independently review and code the focus group transcripts for the themes identified by the author (Irene C. Moore) (30).

## Results

### Focus Groups

Four veterinarian focus groups were held, with four to six participants in each (*n* = 23). Based on information from the 22 participants who filled out the demographic questionnaire, participants ranged in age from 27 to 65 years (mean 44, median 45 years) and had worked as veterinarians between 1 and 40 years (mean 19, median 21 years). Fourteen were female (63%), while eight were male.

Similarly, four RVT focus groups were conducted with four to eight participants per group (*n* = 26). The 26 RVT participants ranged in age from 23 to 50 years (mean 32, median 31 years) and had worked in the field from two to 30 years (mean 9.5, median 8 years). All RVT participants were female.

Intercoder reliability, using percent agreement, was 95% for nominal level themes when coding between the author (Irene C. Moore) and the secondary coders was compared.

### Thematic Analysis

A theme interwoven throughout all veterinarian and RVT groups was Communication – as expressed by one veterinarian, “if there’s not good communication, everything will just fall apart.” Four other themes also repeatedly emerged in all focus groups – Toxic Attitudes and Environment, Work Engagement, Coordination, and Leadership. A number of subthemes were found under each of the major themes. The Toxic Attitude and Environment theme was common in all veterinarian and RVT focus groups. The presence of people exhibiting a toxic attitude, or the presence of a toxic work environment, was thought by all veterinarian and RVT focus groups to have a pronounced impact on the success of the team.

#### Toxic Attitude

A toxic attitude manifested in many forms. For instance, participants described toxic attitudes as disrespect, resistance to change, or avoidance of conflict. Several manifestations of toxic attitudes were frequently mentioned in both the veterinarian and RVT groups, including a lack of motivation, a chronically negative demeanor, always wanting to be the “go to person,” and incompatible personalities (Figure 1).

##### That’s Not My Job

A very common frustration expressed within both the RVT and veterinarian focus groups was dealing with people having a “that’s not my job” attitude. Many comments were related to people not wanting to answer phones, clean kennels, hold animals, or do laundry. These comments were sometimes directed at veterinarians, but often at receptionists or new staff. When people refused to perform certain tasks that would reasonably be considered part of their duties, others felt they were exhibiting a toxic attitude. For instance, RVTs in several focus groups were frustrated with veterinarians who refused to answer the phone or see clients during lunch breaks, and with receptionists who did not want to talk to veterinarians directly. When people refused to assist with these duties or tasks, other staff members often considered this a control or ego issue. The participants felt that people with this attitude were not contributing properly as team members.

In some cases, the “that’s not my job” sentiment was believed to be the result of the receiver of this message not appreciating or understanding what the other person was doing, often because this was not communicated properly. Many veterinarian and RVT groups cited examples where people appeared to be unwilling to help, but they were actually busy with other duties. For instance, communication breakdowns sometimes occurred between the “front” and “back” of the clinic, when people were unaware of what other employees were doing (e.g., employees busy in the reception area, with the perception that employees performing treatments or assisting with diagnostic tests were idle). Communication breakdowns also occurred if individuals perceived jobs were left for them to do, not appreciating that others may have been too busy to complete these tasks.

A number of participants in both the veterinarian and RVT focus groups found that having written job descriptions and consistent training helped alleviate the “that’s not my job” sentiment. However, they indicated it was still important to have accountability and flexibility. As one RVT expressed it “you know there’s some structure to it, you know who’s responsible for what… people are not willing to pull the ‘that’s not my job’ phrase. Everybody is still willing to go that extra mile.”

##### Mood Polluters

A toxic attitude might be a temporary situation. Some RVT groups mentioned that a negative interaction with a client or coworker can color the rest of the day: “You’ll talk to one angry client and whether or not they are justifiably angry is a whole other issue, but they just get you riled up and they get your blood pressure up and they have you on the defensive and then someone says ‘oh well there’s an error on this’. And you’re like, ‘I don’t care, it’s not my problem right now’. It affects how you deal with everybody else that day. If it happens first thing at the beginning of your shift, it can color your whole shift and how you relate to everybody.” People who do this regularly were called “mood polluters” by one RVT. Other participants in the same group acknowledged when people felt comfortable enough with their teams to share these feelings, it brought the team closer together, as they knew other team members would empathize with their experiences.

Several RVT groups discussed the impact chronically negative coworkers can have on the team. A few RVTs mentioned that some of these people were “burned out,” and that “just a couple of people can cause a team to be totally disrupted.” Another RVT indicated these employees repeatedly, “are the ones that leave at 5:01 when the next car is coming in” rather than staying to help out at the end of the day to ensure everyone can get home relatively early.

Participants felt having a mood polluter in the clinic sometimes helped the others unite as a team. One RVT described the situation in her clinic where the veterinarian was on an “emotional roller coaster” on a daily basis. The technician indicated it “really built a strong team below her because we know that as a team, we have to function regardless of the day she’s having.”

The veterinarian groups also indicated that a negative person brings down the whole team. Interestingly, most veterinarian focus groups very quickly moved to talk about how important it was to get rid of mood polluters. Several veterinarian participants mentioned trying to change people’s behavior, but were unsuccessful; thus, they now primarily hire for attitude rather than for specific skills. Although the consensus seemed to be that negative people should be off the team, occasionally veterinarians were hesitant to dismiss those who were in dire need of the job or were long-term members of the community. Nonetheless, several veterinarians also indicated that once they had dismissed a negative person, the clinic atmosphere and efficiency improved.

##### Wanting to be the “Go To” Person

This was a theme particularly prevalent in the veterinarian groups. While the individual exhibiting this behavior would be unlikely to consider it toxic, other team members often considered this trait frustrating. As one RVT put it, “I find there’s a bit of a breakdown in communication when one person wants to stay in charge, so they just don’t tell anybody what’s going on because then they’re the ‘go to’ person.” Some participants felt this was related to wanting to be in a position of power, in that knowledge is power. They indicated a particular problem occurs when the team member who wants to be the “go to” person does not have the appropriate skills or training. For instance, an RVT’s clinic manager had limited veterinary background which impeded her ability to communicate with suppliers. Her coworkers were frustrated because the manager “tells [suppliers] things on the phone and they don’t understand what she’s saying and she doesn’t know what they’re saying and instead of getting us to do it, she just does it herself.”

Other veterinarian and RVT participants acknowledged that they wanted to or expected to be the “go to” person. Some people exhibiting this behavior simply felt that their experience and background best qualified them to do the job. Other participants avoided delegating because they were not confident the task would be done to their expectations.

An observed contrast between the veterinarian and the RVT focus groups was related to people wanting to be acknowledged when they were assigned to be the “go to person.” Many RVT participants were frustrated that they were expected to perform managerial-type duties or be the “head technician” without formally receiving the title. They felt it led to resentment or irritation in the other team members, as their coworkers felt they were shirking their technician duties in order to perform managerial duties. Some also felt it was unfair that they were expected to perform managerial duties without compensation. Several veterinarian groups, on the other hand, felt that “elevating” one of the team members to a different role led to resentment. As one veterinarian articulated, “So we’ve got four technicians, one technician who we kind of call ‘head’ technician, but we do it quietly so we don’t get the others upset.” Another stated, “We have four technicians, but no one is actually the head technician ‘cause we have had a problem with that.”

##### Personality Issues

Many participants in the veterinarian focus groups extensively discussed the impact of people’s personalities on the team. Several veterinarians indicated they would hire primarily for personalities, as they felt they could teach the technical skills, but not change the personalities. They also described that the employee’s personality must fit with the rest of the team. Many clinics had either working interviews or other means of allowing the staff input into hiring decisions. They felt this helped ensure personality compatibility in the team. As one veterinarian put it, “I’m going to make it really clear to you; I’m not firing eight people before I fire you. You have to fit in or it’s not going to work.”

#### Toxic Environment

A toxic environment was described as resulting when “broken communication and tension between staff members” occurred because of underlying issues, including employees lacking the requisite confidence, skills, or knowledge; employees not feeling appreciated; difficulties coping with turnover; and dealing with conflicting demands. It also occurred when conflict was mishandled, or when chronically negative or hostile people were not held accountable for their behavior. The interrelationships of factors contributing to a toxic environment are illustrated in Figure 1.

##### Lacking Confidence, Skills, or Knowledge

All veterinarian and RVT focus groups discussed the impact of people lacking confidence, skills, or knowledge on the rest of the team. People who felt that their coworkers lacked these attributes lost trust in their abilities. A number of RVTs sometimes felt it was necessary to check on their coworkers, leading to frustration by both parties. A few RVTs mentioned they lacked confidence in abilities of new veterinarians or RVTs, questioning their decisions and diagnostic results. In other cases, there was annoyance with new veterinarians when “they are [too] cautious and can’t make a decision.” The RVT groups also were annoyed with coworkers who did not understand or appreciate their skills and knowledge. They cited several examples where on-the-job-trained individuals or people in management positions without veterinary backgrounds did not acknowledge the depth or breadth of RVTs’ skills. In most of the veterinarian groups, no clear differentiation was made between the abilities of RVTs and other members of the veterinary team.

In all of the veterinarian and RVT focus groups, when employees were felt to be lacking in confidence, skills, or knowledge, negative repercussions for the team were believed to result. An outcome in some cases was employees being disrespectful to each other. This was believed to be more commonly observed between different team functions. A group of employees were more likely to be disrespectful to another group of employees (e.g., receptionists to RVTs; veterinarians to RVTs) rather than to members within their own group. For example, one RVT indicated that the receptionists “would never say to the veterinarian ‘look your appointment’s here, let’s get going.’ They sure as hell would say that to us.” In other cases, the toxicity was more subtle: staff would avoid addressing the problem altogether. One veterinarian described the situation in his clinic where an employee repeatedly reported a negative environment, but was reluctant to specifically identify the instigator. The underlying problem for both veterinarian and RVT participants was that a toxic attitude/environment appeared to be present, but there was reluctance to address it.

##### Not Feeling Appreciated

Not feeling appreciated by clients, by veterinarians (in the case of RVTs), or by coworkers contributed to a toxic environment. This theme was much stronger in the RVT focus groups. In some cases, there was reluctance to express this need explicitly. One veterinarian mentioned it required a third party to identify the problem in his clinic. The consultant indicated that his veterinary team did not feel they were appreciated. The veterinarian was surprised at this finding, but realized he had to alter his perception: “you feel like you’re supporting them a fair amount, but if they’re not getting that impression then we need to do more.” The basic problem described in both veterinarian and RVT focus groups was a lack of awareness of what other people do, although it was also tied in with respect or lack of respect for people’s capabilities. All RVT groups expressed frustration that their education, knowledge, and skills were often not recognized. Specifically, at the clinic level they were resentful when on-the-job trained staff received the same pay and performed the same duties that they did, potentially creating a toxic work environment.

Both the veterinarian and RVT group participants described ways of showing appreciation. These included providing small prizes for “catching people doing something right,” reading thank you letters from clients during staff meetings, and encouraging people to provide positive feedback to other coworkers. Some clinics had appreciation events such as parties and team excursions.

##### Coping with Turnover

Many veterinarian and RVT focus groups discussed the challenges to the team environment encountered when dealing with turnover. A negative environment sometimes resulted when a new member joined an already established team as a result of clinic expansion, or to replace people who were leaving or going on leave (e.g., maternity leave). Some RVT groups indicated that staff changes can be stressful for the permanent employees. In one RVT group, staff preferred not to grow: “we don’t want to get bigger but you have to get bigger because we have the demand for it.” Veterinarian and RVT participants indicated challenges with turnover can be related to resistance to change, which was commonly mentioned in both the veterinarian and RVT focus groups. One RVT who was initially hesitant to delegate to others said that she was set in her ways after 20 years, and admitted she was resistant to change; however, once she actually gave up responsibilities to others, it was beneficial for her and the team.

##### Changing the Rules

Several RVT groups indicated a toxic environment may occur when employees are following official procedures, but then aren’t backed up by the veterinarian or by the administration. For example, a client made a request against clinic policy. The RVT communicated the policy to the client, but the veterinarian subsequently ignored the policy and allowed the client their request. As one RVT stated: “That undermines any credibility that you may have developed with the client. That’s not teamwork.” Several RVT participants indicated that this problem can be rectified if the veterinarian clearly lets the client know that an exception is being made.

##### Lack of Consequences

Both RVT and veterinarian focus groups cited examples of a particularly toxic attitude by one or several individuals creating a toxic, hostile clinic environment, especially when there were no consequences to this behavior. For instance, an RVT spoke of her experience working in a clinic with “people fighting all the time” and “hating each other” with no repercussions from management. A recently graduated veterinarian gave the example of a technician who would cross out treatments that she did not agree with, without consequences to this behavior, although the technician was a relatively new graduate herself. This caused interpersonal tensions in the clinic. Another veterinarian from a large clinic described a situation in which a number of people complained about a technician with a very negative attitude, who was shuffled from one department to another, rather than being disciplined or dismissed. Participants indicated this lack of consequences for bad behavior decreased motivation in other clinic staff.

##### Having Unreasonable Expectations

Registered Veterinary Technician and veterinarian participants indicated a toxic environment could occur if people were expected to perform tasks beyond their scope of practice, or tasks that were unrealistic given personnel or facility limitations. This tended to be more of an issue identified within the RVT groups. For instance, in one participant’s practice technicians were expected to “police” the veterinarians in case they prescribed the wrong medication: “The [clinic] owner will come in and ask, ‘why isn’t this renal failure cat on potassium?’ ‘It’s because the [other] doctor didn’t call for it.’ ‘Well you guys should have caught that and asked why the doctor didn’t call for potassium.’” In other cases, each veterinarian in a multiple veterinarian practice had preferred methods of doing things, which led one technician to lament: “Everybody does things differently and they expect you to remember that. Some days I remember where I said, ‘I can’t work here anymore this place is driving me crazy’ because there was such a lack of communication and high expectations.” While people in most veterinary clinics are expected to multi-task, participants felt if people were expected to take on too many tasks simultaneously, client service and potentially even animal safety may be compromised. An extreme example of this was exemplified by one RVT’s experience: “…for about 3 months I was the only employee… I was monitoring surgery, setting up the surgery, answering the phone while cashing people out for [pet] food…. It’s just not effective; it’s not even cost effective.” Feeling overloaded thus led to a toxic environment for some participants, as they felt they could not provide the level of client and patient service they wanted to or were expected to. Veterinarian participants described unreasonable expectations in terms of having conflicting demands. For instance, one veterinarian indicated, “it would be nice sometimes to be able to do my vet stuff and not have to focus on taking an x-ray because I don’t want to be doing that. I need to be researching things…it’s just not an option right now.”

##### Conflicting Demands

Conflicting demands created a toxic environment according to most participants in both the veterinarian and RVT focus groups. For the RVTs, problems tended to occur when people received consistently conflicting messages from two or more different people, leaving them unsure about what they should do. One RVT described the situation at her clinic: “You get the son telling you one thing and the father tells you something else. Or the office manager, who is the spouse, telling you something else. Then you have to precariously balance the beam.” Conflicting demands were identified as occurring when the clinic is very busy or understaffed, resulting in people not knowing which situations should be handled first. RVT and veterinarian participants mentioned the problem of conflicting demands was exacerbated when people are unaware of what others are doing. In the latter situation, they indicated resentment may build up when a group of people are extremely busy trying to manage conflicting demands and perceive others are not doing very much.

In the veterinarian groups, several mentioned the conflict they felt juggling management and clinical duties. In some cases, it was also related to trying to having a work/life balance, which was sometimes difficult to achieve. As one veterinarian stated: “As owners you could be a good vet but not a good manager, that’s what I feel. It’s very difficult to combine those two together, there is a compromise. Either you could be a good vet and a good manager, but then you are not a good spouse or a family man.” Multiple competing demands sometimes created a toxic environment in the clinic, as it resulted in responsibilities not receiving the attention they deserved.

##### Lack of Leadership

Lack of leadership, whether due to absentee owners/managers, or ineffective leaders was a source of frustration leading to resentment or confusion in team members. Several RVT groups mentioned that the owners of their clinics came only for limited times each week, so that “favorite clients” could see them. Another RVT complained that: “The owner is there in body sometimes and never there in mind so it’s the 2 managers that do everything from business to the bottom end.”

Occasionally people want to provide leadership, but are not effective at it, as was expressed by one technician: “We have two owners… One doesn’t want to be a manager but she has to do some of it. The other one wants to manage but isn’t great at it.”

The veterinarian groups did not specifically talk about absentee owners, although many did speak about wanting to achieve a work/life balance and managing expectations. Others spoke about potential problems with multiple owners or a lack of structure, which may result in frustration and confusion. As one associate indicated, “The leaders are not there, so that I’m lacking the leader and that doesn’t make for a good team …I can’t step up and be the leader. I don’t make decisions for the practice ‘cause it’s not my practice.”

In a clinic with a toxic atmosphere, some veterinarian and RVT participants felt that having a more formal organizational structure might improve the situation. Others felt that the problem was “more to do with personality than actual job description – and ego.”

## Discussion

The impact people with a toxic attitude had on the overall functioning of the veterinary team was a very common theme in both the veterinarian and RVT groups. Participants indicated the presence of a toxic environment also had a negative effect on employees. This is congruent with research in human healthcare, as lack of collaboration among healthcare professionals, ineffective management, and work stress are associated with voluntary turnover intention and job dissatisfaction (16, 31–33). Thus, it behooves veterinary owners and managers to assess the presence of a toxic environment and to address factors contributing to it.

Toxic attitudes and a toxic environment can lead to both relationship conflicts (e.g., values and interpersonal style) and task conflicts (e.g., procedures, policies, and distribution of resources) (34). A meta-analysis of associations between relationship conflict, task conflict, team performance, and team member satisfaction found relationship and task conflicts to be negatively associated with both team satisfaction and team performance (34). The types of negative behaviors considered problematic in both the veterinarian and RVT groups are consistent with those identified in other working groups across many organizations. These behaviors include leaving tasks for other people to complete, being persistently pessimistic, being excessively critical of others, and demeaning fellow team members. Both veterinarian and RVT groups cited frequent examples of individuals not completing their tasks. The “that’s not my job” subtheme exemplifies this toxic attitude and caused distress for some participants. To alleviate problems with tasking being left undone, several participants in the current study felt clinics could provide detailed job descriptions, but also clarify the necessity of assisting other team members when required.

The focus groups indicated frustration with chronically negative people exhibiting persistently pessimistic attitudes and behaviors. According to Furr and Funder (35), these negative people may also be more likely to express irritation, anxiety, and insecurity. In the veterinarian and RVT focus groups, these are the “mood polluters” that create negativity in the workplace, affecting the emotions, moods, and attitudes of the rest of the team in a disproportionate manner. Research shows people give more credence to negative emotional information and dwell on negative events more often and for a longer period of time than they do on positive events (36, 37). Some focus group members found ways to encourage positivity in their practices through team-building activities and acknowledgment of positive team work.

When people violate important interpersonal norms, they may be referred to as “interpersonal deviants” (38, 39). In the focus groups, the “deviant” behaviors most likely to appear were making hurtful comments, behaving rudely, or embarrassing people in front of others. Research in other professions has shown that it is critical that these behaviors not be tolerated, as it results in distrust of the deviant team member by the rest of the team (40) and causes time to be wasted by team members distracted by the negative behaviors (41). To alleviate problems created by instigators of incivility, veterinary owners and managers could improve orientation and training of new employees, set zero-tolerance expectations for incivility, document and address instances of these behaviors, and avoid hiring anyone that has exhibited these behaviors in the past (41, 42).

The type of behavior characterized by people wanting to be the “go to” person does not fit as well with the above categories of negative behaviors. Instead, they would more appropriately be described as “constructive deviants” (43). While these people may be perceived to negatively impact the team, in some cases they may not disturb the group’s effectiveness. Instead, particularly if they do have the requisite skills and knowledge, they help the team achieve its goals. Nonetheless, if the rest of the team is impacted by this behavior, it needs to be addressed by outlining expectations and showing the benefits of sharing responsibilities and tasks with coworkers.

The issue of personalities was discussed extensively, particularly in the veterinarian groups. While there has been extensive research on the impact of personalities in the workplace, no generally accepted taxonomy of personality exists (44, 45). Furthermore, the relationship between personality testing and occupational performance is controversial (46). While there seems to be general agreement that the underlying personality or temperaments are related to core needs and values, and do not change over time, people do have control over their behaviors. Thus, rather than selecting for certain personality traits, it might be more effective to capitalize on the positive attributes each personality type brings to the workplace. It would also be prudent to set out firm guidelines for appropriate workplace behavior. The working interviews described by some participants, as well as the use of behavioral interviews and careful screening of references for potential new hires, may ensure compatibility with the team. The selection process can also be designed to select for flexible and adaptable employees.

Participants indicated a toxic environment may occur when teammates lack confidence, skills, or knowledge, or because teammates are exhibiting interpersonal deviant behaviors. This can also be tied in with people not feeling appreciated, and can lead to distrust and a lack of respect. Distrust can distract from task performance, which may impair team outcomes (30, 47, 48). In addition, lack of respect frequently manifests as a lack of civility. Incivility often leads to distraction: people spend time worrying about the incident and discussing it with others (41). It may also lead to turnover; in a study of individuals that experienced incivility in the workplace, up to 50% considered leaving their positions, with 12% actually leaving (41). Feeling respected and respecting others are keys to cooperation (49). When people see themselves as individuals rather than part of a team, they may think and act more selfishly, rather than working cooperatively (50). Thus, by addressing incivility, veterinary clinics can potentially reduce unproductive time as well as reducing turnover. Reviewing hiring and training practices and diligent monitoring and recording of problems will help prevent and curtail workplace incivility.

In many cases, the presence of people with a negative attitude leads to a toxic environment, particularly if the negative behavior is not addressed. Responses to negative behavior may include attempting to change the person’s behavior, removing the person from the workplace, or protecting oneself through defensive behaviors (40). When motivating the employee or removing the person from the group is not possible, more severe manifestations of a toxic environment may result. In response to a negative person, other employees may become defensive in order to “protect and repair one’s own sense of autonomy, status, self-esteem, or wellbeing” [(40) (p. 187)]. These defensive behaviors may include “lashing out, revenge, unrealistic appraisals, distraction, various attempts at mood maintenance, and withdrawal” [(40) (p. 187)]. The actively hostile environments described in some of the focus groups may have been defensive behaviors in response to unaddressed negative behaviors. According to the literature, dealing with these behaviors is a leadership responsibility. If leaders do not attend to the situation, the result is dissatisfaction with the team (51). Thus, it behooves the veterinary profession to not ignore negative behaviors. Rather, the perpetrators should be confronted, given the opportunity to correct the behavior, and given consequences if their behavior does not change. Veterinarians and other clinic leaders may need additional training in communication and leadership skills in order to address negative behaviors effectively.

Having an excessive workload may result in people feeling the environment is toxic, as they may feel there are unreasonable expectations. A number of articles describe the impact of this problem in the human healthcare field. Aiken, Clarke, and Sloane (52) found that job dissatisfaction, burnout, and perceived quality of care were significantly related to hospital staffing levels. Hospitals with Magnet certification are characterized by higher nurse staffing levels and empowerment of nursing staff. Studies have shown that in Magnet hospitals, patient satisfaction is higher and nurses report less burnout, less intention to leave, and more manageable workloads (53–55).

A toxic environment was also described when there were conflicting demands in the workplace. While potentially related to communication, conflicting demands were also a result of a clinic being extremely busy or understaffed. Many participants found it difficult to cope when the clinic was short-staffed. It may be prudent for veterinary clinics to examine staffing ratios and also ensure employees are empowered to do as much as they are legally allowed to do. This may have financial advantages as well; according to an Ontario Veterinary Medical Association study, clinics with higher non-DVM to DVM ratios (from 2.9 to 4.2 per full time equivalent DVM) function more effectively through a higher net practice income than clinics with lower ratios (56). Given the concern about work/life balance expressed by a number of participants, it behooves managers to review work hours and staffing levels to ensure team members are not feeling overwhelmed.

Focus group research is designed to explore participants’ perspectives and attitudes about various topics (24–27). The participants were volunteers from a limited geographic area. Participants included only veterinarians and RVTs, thus comments may not be reflective of other team members. In addition, participants may not be reflective of all RVTs or veterinarians, as they might have had a greater interest in and stronger opinions about veterinary healthcare teams than non-participants. Furthermore, qualitative research is not intended to be generalized to all individuals. Nonetheless, veterinary teams can use the findings to reflect on whether the views represent their own situations and how they can maximize team effectiveness within their own practice environments.

Further qualitative and quantitative research studies in this area are warranted. In particular, intervention studies could determine whether team effectiveness can be improved through education and training of team members, or by addressing specific factors within a practice found to be contributing to a negative environment. Future research efforts should also focus on the impact of team effectiveness on other outcomes at the client, clinic, and patient level, including client satisfaction and adherence, clinic turnover and profitability, and medical errors.

## Author Contributions

IM is the corresponding author, and did the research and writing of the article as part of a Master’s thesis. JC was IM’s graduate supervisor, and was extensively involved throughout all phases of the research and writing of the article. CA, PC, and JS were all graduate committee members, and provided feedback in developing the research proposal, critiqued aspects of the data analysis, and aided in revisions of the paper. All authors have given approval for the article submitted for publication.

## Conflict of Interest Statement

The authors declare that the research was conducted in the absence of any commercial or financial relationships that could be construed as a potential conflict of interest.
