# Supplementary material for: Exploring the Impact of Toxic Attitudes and a Toxic Environment on the Veterinary Healthcare Team
Source: Front Vet Sci. 2015 Dec 23;2:78. doi: 10.3389/fvets.2015.00078 (PMC4688347; doi:10.3389/fvets.2015.00078)
Supplement: Supplementary file 1 [file Data_Sheet_1.DOCX]

**Appendix I**

**Open-ended Questions and follow-up probes used during the focus group discussions involving veterinarians:**

| **Discussion topic** | **Key questions and follow-up probes** |
| --- | --- |
| Characteristics of effective teams | Thinking of a team you’ve been on that has functioned well (e.g., work-related, volunteer-related or sports-related team). What are the characteristics of a team that functions well?  Probes:   - *How does that contribute to the functioning of an effective team?* - *What makes that an important part of a high functioning team?* - *Does this apply to the functioning of every team?* - *Does this apply to veterinary teams? (How? To what effect?)* |
| Veterinarians role on team | How do you see your role as part of the veterinary health care team?   - - As a veterinarian is the role different for associates vs practice owners?   - As a veterinarian is the role different for practice owners vs partners in a practice?   - Do you think this is a common role/ experience among veterinarians in practice?   - Do you think other members of the team perceive a veterinarians role in this same way? |
|  |  |
| Other team members’ roles | How do you see the role of other members of your practice team?  Prompts (Technician):   - - What are the roles of veterinary technicians within your current practice team?   - How are veterinary technicians utilized in your current practice team?   - How does this contribute to the effectiveness of your practice?   - What relationship do veterinary technicians have with you? Other members of the team?   Probes:   - *How does that role contribute to the overall effectiveness of your practice?* - *Is that the appropriate role for a veterinary technician? Why or why not?* - *Are there areas veterinary technicians should be used more? Areas used less?*   Prompts (Other members):   - - Who are the other team members within your current practice team?   - What role do they have within your practice team?   - How do they contribute to the effectiveness of your practice?   Probes:   - *How does that contribute to the overall effectiveness of your practice?* - *Should that be the role of that team member? Why or why not?* - *Are there areas where this member should be used more? Areas used less?* |
|  |  |
| Challenges to working as a team | What are the challenges you experience interacting with other members of the veterinary team?  Probes:   - *What do you feel is the underlying cause?* - *How does this affect the overall team? The client? The veterinary patient?* - *Is this a common challenge/ barrier in veterinary practice?* - *How could this be improved?* |

**Appendix II**

**Open-ended Questions and follow-up probes used during the focus group discussions involving Registered Veterinary Technicians:**

| **Discussion topic** | **Key questions and follow-up probes** |
| --- | --- |
| Characteristics of effective teams | Thinking of a team you’ve been on that has functioned well (e.g., work-related, volunteer-related or sports-related team). What are the characteristics of a team that functions well?  Probes:   - *How does that contribute to the functioning of an effective team?* - *What makes that an important part of a high functioning team?* - *Does this apply to the functioning of every team?* - *Does this apply to veterinary teams? (How? To what effect?)* |
| Registered-veterinary technicians role on team | How do you see your role as part of the veterinary health care team?   - - As a Registered Veterinary Technician is the role different for non-registered technicians?   - As a Registered Veterinary Technician is the role different for veterinary assistants?   - Do you think this is a common role/ experience among Registered Veterinary Technicians in practice?   - Do you think other members of the team perceive a Registered Veterinary Technician’s role in this same way? |
|  |  |
| Other team members’ roles | How do you see the role of other members of your practice team?  Prompts (Veterinarians):   - - What are the roles of veterinarians within your current practice team?   - Are they different for associate vs. owner veterinarians?   - Is there a clear definition of which duties are to be performed by veterinarians vs. by registered-veterinary technicians in your practice?   - How does this contribute to the effectiveness of your practice?   - What relationship do veterinarians have with you? Other members of the team?   Probes:   - *How does that role contribute to the overall effectiveness of your practice?* - *Is that the appropriate role for a veterinarian? Why or why not?* - *Are there tasks that would be more appropriately done by veterinary technicians? Others that you are doing that should be done by veterinarians?*   Prompts (Other members):   - - Who are the other team members within your current practice team?   - What role do they have within your practice team?   - How do they contribute to the effectiveness of your practice?   Probes:   - *How does that contribute to the overall effectiveness of your practice?* - *Should that be the role of that team member? Why or why not?* - *Are there areas where this member should be used more? Areas used less?* |
|  |  |
| Challenges to working as a team | What are the challenges you experience interacting with other members of the veterinary team?  Probes:   - *What do you feel is the underlying cause?* - *How does this affect the overall team? The client? The veterinary patient?* - *Is this a common challenge/ barrier in veterinary practice?* - *How could this be improved?* |
